# Hearing Loss, Hearing Aids, and Cognition

**DOI:** 10.1001/jamanetworkopen.2024.36723

**Published:** 2024-10-01

**Authors:** Baptiste Grenier, Claudine Berr, Marcel Goldberg, Xavier Jouven, Marie Zins, Jean-Philippe Empana, Quentin Lisan

**Affiliations:** 1 Université Paris Cité, Inserm, U970, Paris Cardiovascular Research Center, Integrative Epidemiology of Cardiovascular Disease, Paris, France; 2 University of Montpellier, INM, Inserm U1298, Montpellier, France; 3 Université Paris Cité, Population-Based Cohorts Unit, INSERM, Paris Saclay University, UVSQ », UMS 011, Paris, France; 4 Assitance Publique-Hôpitaux de Paris, Department of Cardiology, European Hospital Georges Pompidou, Paris, France; 5 Department of Head and Neck Surgery, Foch Hospital, Suresnes, France

## Abstract

**Importance:**

Most observational studies examining the association between hearing loss (HL) and cognitive impairment used subjective measures of hearing and addressed only one dimension of cognition, and very few investigated the potential benefit of hearing aids (HAs).

**Objective:**

To evaluate objectively measured HL and several dimensions of cognition and estimate the association with HA use.

**Design, Setting, and Participants:**

A cross-sectional analysis of the CONSTANCES cohort study, which recruited participants from January 1, 2012, to December 31, 2020, was conducted. Participants were a representative sample of adults (age, 45-69 years) with audiometric data and cognitive evaluation from 21 preventive health centers in France. Data analysis was conducted from April 1 to September 15, 2023.

**Exposure:**

The main exposure was HL, which was defined by a pure-tone average in the best ear higher than 20 dB hearing level for mild loss and 35 dB hearing level for disabling loss. Secondary exposure was self-reported HA use.

**Main Outcomes and Measures:**

Cognition was evaluated at study inclusion by a standardized battery of 5 cognitive tests conducted by trained neuropsychologists. A global cognitive score was computed from principal component analysis and global cognitive impairment was defined as having a score less than or equal to the 25th percentile of the distribution.

**Results:**

The study population included 62 072 participants with audiometric data (mean [SD] age, 57.4 [7] years; 52% women). Overall, 38% (n = 23 768) had mild HL, 10% (n = 6012) had disabling HL, and 3% (n = 1668) were HA users. In multivariable analyses, mild HL (odds ratio [OR], 1.10; 95% CI, 1.05-1.15) and disabling HL (OR, 1.24; 95% CI, 1.16-1.33) were associated with greater global cognitive impairment. The odds of cognitive impairment did not differ significantly between all participants with HA use and participants with disabling HL without HAs (OR, 0.94; 95% CI, 0.83-1.07), except among participants with depression (OR, 0.62; 95% CI, 0.44-0.88).

**Conclusions and Relevance:**

In this cohort study, an association between the severity of HL and global cognitive impairment was found. The use of HAs was not associated with significantly lower odds of cognitive impairment. The findings suggest that it may be useful to monitor cognitive function in middle-aged individuals with HL.

## Key Points

**Question:**

Is hearing loss associated with an increased risk of cognitive impairment in middle-aged adults?

**Findings:**

In a cohort study of 62 072 participants aged 45 to 69 years, there was an association between the severity of hearing loss objectively measured with audiometric tests and several domains of cognitive impairment. The odds of cognitive impairment did not differ significantly between participants with hearing aid use and participants with disabling hearing loss without hearing aid use.

**Meaning:**

The findings of this study suggest that monitoring of cognitive function in patients with hearing loss may be useful.

## Teaser

This cohort study examines cognitive function in middle-aged adults with hearing loss, both with and without use of hearing aids.

## Introduction

The prevalence of cognitive impairment is increasing, and projections estimate that, by 2050, up to 150 million individuals worldwide will be living with dementia.^1^ Given the major burden of cognitive decline and the absence of curative treatment, identifying modifiable risk factors is of importance. The *Lancet* Commission estimated that hearing loss (HL) was the leading potentially modifiable risk factor for dementia and that 8% of the cases could be attributable to HL.^2^ Hearing loss currently affects more than half of adults aged 60 to 65 years, with the prevalence continuing to increase as the population ages.^3,4^

However, several studies examining the association between HL and cognitive impairment relied on self-reported HL,^5,6,7,8,9,10,11^ which is known to be poorly correlated with objectively measured hearing.^12^ Furthermore, some studies used only 1 test to assess cognitive impairment, whereas cognition is multidimensional.^9,13^ In addition, numerous studies were of small sample size^14,15,16,17,18,19,20,21^ and focused on selected populations, such as participants aged 70 years and older or with preexisting mild cognitive impairment,^11,17,19,22^ given the low prevalence of both cognitive impairment and HL at younger ages. Few studies have addressed HL objectively and evaluated several domains of cognition.^12,13,19,23^

Some studies have claimed that hearing rehabilitation could prevent cognitive decline.^2^ However, most relied on administrative codes of dementia, did not use quantitative cognitive evaluation, or were of limited sample size.^11,24^ Moreover, a recent randomized clinical trial did not demonstrate unequivocal benefit of hearing aid (HA) use on cognition.^25^ In fact, few prior studies have used a relatively large sample size and performed a quantitative evaluation of cognition.^26,27^

The main objective of this study was to investigate the association between HL and cognitive impairment in participants aged 45 to 69 years. The secondary objective was to examine the possible benefit of HA use in cognitive impairment. This analysis used objective assessment of hearing status, extensive cognition evaluation, and a large, nationwide, representative sample of middle-aged French adults.

## Methods

### Study Design

This study used data from the French population-based CONSTANCES cohort. From January 1, 2012, to December 31, 2020, 220 000 adults aged between 18 and 69 years were randomly selected from the National Health Insurance Fund, which covers more than 85% of the French population. A sampling scheme stratified by age, sex, socioeconomic status, and region of France was used to ensure a representative sample of the National Health Insurance. Statistics on racial and ethnic groups is forbidden by law in France. Participants were a representative sample of adults (age, 45-69 years) with audiometric data and cognitive evaluation from 21 preventive health centers in France. Detailed information about the study design has been published elsewhere.^28,29^ All participants provided written informed consent; no financial compensation was provided. The CONSTANCES study followed the Declaration of Helsinki^30^ and received necessary approval from the National Data Protection Authority and the institutional review board of the National Institute for Medical Research. The present study was approved by the Comité d’Evaluation Ethique de l’Inserm. This report follows the Strengthening the Reporting of Observational Studies in Epidemiology (STROBE) reporting guideline.

### HL and HA Use

At study inclusion, all participants underwent a hearing test in either a soundproof testing room or an air-ambient quiet room, based on the recruitment center’s equipment. The air-conduction thresholds were assessed at 0.5, 1, 2, 4, and 8 kHz. All measuring processes were performed in accordance with the International Organization for Standardization 8253-1 standard.^31^ The pure-tone average (PTA) for each ear was calculated as the mean of the 0.5-, 1-, 2-, and 4-kHz frequencies. Hearing status was defined as normal hearing (PTA <20 dB hearing level for the better ear) or HL (PTA ≥20 dB hearing level), categorized as mild HL (PTA 20-34.99 dB hearing level) or disabling HL (PTA ≥35 dB hearing level), according to recommendations.^4,32^ All HL refers to all participants with HL, that is, participants with mild or disabling HL and those with HA. Audiometric testing was not performed for participants using HAs at study inclusion. They were considered to have disabling HL (PTA ≥35 dB hearing level), which corresponds to a suggested threshold.^33^

### Cognitive Assessment

At study inclusion, participants aged 45 years or older underwent an extensive cognitive evaluation. Trained neuropsychologists conducted several cognitive tests: the Digit Symbol Substitution Test (DSST), a subtest of the Wechsler Adult Intelligence Scale–Revised, to evaluate attention, psychomotor speed, and reasoning^34^; the Free and Cued Selective Reminding Test (FCSRT) to evaluate episodic verbal memory^35^; the Trail Making Test (TMT-A and TMT-B) to evaluate shifting abilities (TMT-A) and executive functions (TMT-B)^36^; the Mini-Mental State Examination (MMSE) to evaluate global cognitive functioning^37^; and the Verbal Fluency Tasks (VFT) to evaluate language abilities (semantic and phonemic fluency tasks).^38^

First, to establish a comprehensive measure of cognitive performance (taking into account several cognitive domains), a global cognitive score was computed from principal component analysis including the DSST, FCSRT immediate free recall, and TMT tests, as previously published.^39^ Given that both MMSE and VFT results depend on the auditory understanding of the instructions, these scores were not included in the main global cognitive score but were considered in sensitivity analyses. Other subdomains of the FCSRT (ie, the cued and delayed recalls) were also not considered in the main global cognitive score given their known limited discriminatory power due to a pronounced threshold effect.^35^ The global cognitive score was represented by the first axis of the principal component analysis that accounted for 58% of the variance (eFigure 1 in Supplement 1). Individuals with scores less than or equal to the 25th percentile were classified as having global cognitive impairment, as previously published.^39^ This threshold was defined to identify participants with poorer cognitive performance among this relatively young cohort. Second, each cognitive test was considered separately, and cognitive impairment was defined by scores less than or equal to the 25th percentile of the distribution or greater than or equal to the 75th percentile for the TMT, using established norms from the CONSTANCES cohort, adjusted on age, sex, and educational level.^40^ Third, in sensitivity analyses, a second global cognitive score was created from principal component analysis considering all available cognitive tests.

### Covariates

Body mass index was calculated as weight in kilograms divided by height in meters squared. Diabetes was self-reported. Hypertension was defined as self-reported treated hypertension or a measured systolic and diastolic blood pressure greater than 140/90 mm Hg. Depression was defined as a Center for Epidemiologic Studies Depression Scale score of 19 or higher, according to the validated cutoff of the French version.^41^ Prevalent cardiovascular diseases (CVDs) were defined as a self-reported personal history of myocardial infarction, angina, stroke, or peripheral arterial disease. Contextual social deprivation was estimated using the French Deprivation Index, a composite score derived from mean household income, educational level, type of profession, and average unemployment rate at the level of their residential commune (municipality).^39^ Participants within the fifth quintile were defined as presenting the most-deprived environment.^39,42^ Individual deprivation, evaluated using the Evaluation of Deprivation and Inequalities in Health Screening Centres (EPICES) score, an 11-item instrument designed to evaluate precarious living conditions, such as housing, health, recreational opportunities, social connections, and financial challenges. A participant was considered deprived when the EPICES score was greater than or equal to 30.17.^43^ Noise exposure at work was self-reported based on the following question: “Do you work (or have you worked) in an environment where you needed to raise your voice to speak to someone located less than 2 meters from you? Or with or near noisy machinery or vehicles?” Smoking was self-reported and categorized as never, former, and current smoker. Educational level was assessed using the 2011 International Standard Classification of Education and categorized as primary education, lower secondary education, upper secondary education, bachelor’s degree or equivalent, and master’s or doctoral degrees.^44^

### Statistical Analysis

Data analysis was conducted from April 1 to September 15, 2023. Level of statistical significance was set at *P* < .05, and hypothesis tests were 2-sided. The association between hearing status (normal, mild HL, and disabling HL) and global cognitive impairment was explored in multivariable logistic regression. Odds ratios (ORs) and their 95% CIs were adjusted for age, sex, and educational level (model 1), and then for all the remaining covariates described previously (model 2), which were selected based on published literature.^2,3^ This analysis was rerun considering each main cognitive score separately (ie, DSST, FCSRT, and TMT) to assess specific domains of cognitive impairment. Then, we evaluated the association between HA use and cognition. The eligible population for this analysis comprised participants with disabling HL without HAs and participants with HAs. To limit indication bias, propensity score (PS) analyses were performed. We calculated the propensity of using HAs in a logistic regression model adjusting for the following variables: sex, age, body mass index, personal and social deprivation, educational level, noise exposure, diabetes, hypertension, prevalent CVD, depression, smoking, and alcohol consumption. Then, the association between HA use and global cognition impairment was quantified after (1) adjusting for PS, (2) PS matching (1:1), and (3) PS-weighted analysis (inverse probability of treatment weighting).^45^

Several sensitivity analyses were performed to assess the robustness of the findings. First, we assessed the association between HL and MMSE and VFT scores, and 3 subtests of the FCSRT, together with a second global cognitive score including all available cognitive scores. Second, we reevaluated the association of HL and HA use with cognition in the following contexts: (1) considering the global cognitive score as a continuous variable using multivariable linear regression analysis, (2) stratifying the analysis by depression status given the potential confounding effect of depression on cognition,^2,3,46^ and (3) using multivariable imputation by chained equations to account for missing data.^47^ Third, main analyses were rerun with adjustment on hearing test condition (soundproof or air-ambient testing). All analyses were performed using R software, version 4.3.1 (R Foundation for Statistical Computing).

## Results

### Study Population

Of the 85 885 participants aged 45 to 69 years at study recruitment, 4597 had missing audiometric data and 4074 were missing cognitive evaluation. After excluding participants with missing covariates (n = 15 142), 62 072 participants were included in the present study (eFigure 2 in Supplement 1). Compared with excluded participants, those included were more frequently women, younger, less often deprived, and had higher educational levels, fewer comorbidities, and less depressive symptoms. The population was mostly individuals of White race (eTable 1 in Supplement 1).

The mean (SD) participant age was 57.4 (7) years, 52% were women, and 48% were men. Overall, 49% of participants (n = 30 624) had normal hearing (mean [SD], PTA 14.2 [4] dB hearing level in the better ear), 38% (n = 23 768) had mild HL (mean [SD], PTA 26.6 [4] dB hearing level), 10% (n = 6012) had disabling HL without HA use (mean [SD], PTA, 45.0 [10] dB hearing level), and 3% (n = 1668) were HA users. The characteristics of the study participants by hearing status are presented in Table 1. Participants with poorer hearing performances were more frequently men, older, with more frequent social and personal deprivation, lower educational level, higher body mass index, more comorbidities, and more often exposed to noise at work.

**Table 1.** Participants’ Characteristics by Hearing Status

Characteristic	No. (%)					
	Entire population (N = 62 072)	Normal hearing (n = 30 624)	HL			
			All HL (n = 31 448)	Mild HL (n = 23 768)	Disabling HL without HA (n = 6012)	Hearing aid users (n = 1668)
Pure-tone average, mean (SD), dB hearing level	22.1 (11)	14.2 (4)	30.3 (9)	26.6 (4)	45.0 (10)	NA
Sex						
Male	29 832 (48)	13 541 (44)	16 291 (52)	11 823 (50)	3572 (59)	896 (54)
Female	32 240 (52)	17 083 (56)	15 157 (48)	11 945 (50)	2240 (41)	772 (46)
Age, mean (SD), y	57.4 (7)	54.9 (7)	59.9 (7)	59.3 (7)	61.6 (6)	62.3 (6)
BMI, mean (SD)	25.7 (4)	25.2 (4)	26.1 (4)	26.0 (4)	26.6 (5)	26.2 (4)
Noise exposure	18 682 (30)	8276 (27)	10 406 (33)	7481 (31)	2289 (38)	636 (38)
Social deprivation	12 129 (20)	5616 (18)	6513 (21)	4863 (20)	1296 (22)	354 (21)
Personal deprivation	8652 (14)	3862 (13)	4790 (15)	3447 (15)	1119 (19)	224 (13)
Educational level						
Primary	1617 (3)	611 (2)	1006 (3)	707 (3)	253 (4)	46 (3)
Lower secondary	4795 (8)	1754 (6)	3041 (10)	2082 (9)	794 (13)	165 (10)
Upper secondary	24 097 (39)	10 580 (35)	13 517 (43)	10 021 (42)	2808 (47)	688 (41)
Bachelor’s degree	20 413 (33)	11 185 (37)	9228 (29)	7328 (31)	1404 (23)	496 (30)
Master’s or doctoral degree	11 150 (18)	6494 (21)	4656 (15)	3630 (15)	753 (13)	273 (16)
Comorbidities						
Diabetes	2272 (4)	760 (2)	1512 (5)	1047 (4)	353 (6)	112 (7)
Prevalent CVD	2150 (3)	668 (2)	1482 (5)	936 (4)	388 (6)	108 (6)
Hypertension	11 183 (18)	4268 (14)	6915 (22)	4938 (21)	1541 (26)	436 (26)
Depression	7765 (13)	3777 (12)	3988 (13)	2958 (12)	805 (13)	225 (13)
Smoking at baseline						
Never	27 688 (45)	14 224 (46)	13 464 (43)	10 283 (43)	2452 (41)	729 (44)
Past	26 384 (43)	12 292 (40)	14 092 (45)	10 450 (44)	2841 (47)	801 (48)
Current	8000 (13)	4108 (13)	3892 (12)	3035 (13)	719 (12)	138 (8)
Alcohol consumption						
Never	2447 (4)	1186 (4)	1261 (4)	920 (4)	267 (4)	74 (4)
Low risk	49 113 (79)	24 214 (79)	24 899 (79)	18 857 (79)	4725 (79)	1317 (79)
Harmful	8371 (13)	4175 (14)	4196 (13)	3174 (13)	802 (13)	220 (13)
Dependence	2141 (3)	1049 (3)	1092 (3)	817 (3)	218 (4)	57 (3)

Abbreviations: BMI, body mass index (calculated as weight in kilograms divided by height in meters squared); CVD, cardiovascular disease; HA, hearing aid; HL, hearing loss; NA, not applicable.

### Distribution of Cognition Score by Hearing Status

As presented in Table 2, the proportion of global cognitive impairment increased with the level of HL among participants with normal hearing (16%), mild HL (27%), and disabling HL (37%) (*P* < .001 for trend). This trend was observed at all ages from 45 to 69 years (eFigure 3 in Supplement 1). A similar trend was observed for each cognitive test (Table 2).

**Table 2.** Distribution of Participants’ Cognitive Performances by Hearing Status^a^

Measure	Entire population (N = 62 072)	Normal hearing (n = 30 624)	HL			
			All HL (n = 31 448)	Mild HL (n = 23 768)	Disabling HL without HA (n = 6012)	Hearing aid users (n = 1668)
**Global cognition score**						
Score, mean (SD)	0.09 (1.5)	0.39 (1.4)	−0.21 (1.5)	−0.11 (1.5)	−0.54 (1.6)	−0.38 (1.5)
Impaired, No. (%)	14 233 (23)	5028 (16)	9205 (29)	6389 (27)	2249 (37)	567 (34)
**Digit Symbol Substitution Test**						
Score, mean (SD)	66.7 (14)	69.5 (14)	63.9 (14)	64.7 (14)	61.0 (14)	62.6 (14)
Impaired, No. (%)	15 006 (24)	6906 (23)	8100 (26)	5961 (25)	1721 (29)	418 (25)
**Free and Cued Selective Reminding Test**						
Score, mean (SD)	32.5 (6)	33.3 (5)	31.7 (6)	31.9 (6)	30.8 (6)	31.2 (6)
Impaired, No. (%)	17 438 (28)	8334 (27)	9104 (29)	6805 (29)	1942 (30)	477 (29)
**TMT score**						
TMT-A score, mean (SD)	33.3 (12)	31.8 (11)	34.8 (12)	34.3 (12)	36.5 (13)	36.3 (13)
TMT-A impaired, No. (%)	14 678 (24)	7025 (23)	7653 (24)	5617 (24)	1615 (27)	421 (25)
TMT-B, mean (SD)	65.8 (29)	61.6 (26)	70.0 (31)	68.5 (30)	75.3 (35)	72.1 (32)
TMT-B impaired, No. (%)	14 436 (23)	6784 (22)	7652 (24)	5663 (24)	1594 (27)	395 (24)

Abbreviations: HL, hearing loss; TMT-A, Trail Making Test to evaluate shifting abilities; TMT-B, Trail Making Test to evaluate executive functions.

^a^ Impairment was defined by a score less than or equal to 25% of the total population’s score for global cognitive impairment and by a score less than or equal to 25% of the norms defined in the CONSTANCES cohort, adjusted for sex, age, and educational level for the Digit Symbol Substitution Test and Free and Cued Selective Reminding Test, and greater than or equal to 75% for the TMT.

### Association Between Hearing Status and Cognitive Impairment

In model 1, mild HL (OR, 1.13; 95% CI, 1.07-1.18) and disabling HL (OR 1.32; 95% CI, 1.23-1.41) were related to global cognitive impairment. As shown in the Figure, associations were minimally attenuated following adjustment for additional covariates (model 2), with ORs of 1.10 (95% CI, 1.05-1.15) for mild HL and 1.24 (95% CI, 1.16-1.33) for disabling HL. In these analyses, older age, male sex, noise exposure at work, individual deprivation, diabetes, prevalent CVD, hypertension, depression, and lower level of education were associated with global cognitive impairment (eTable 2 in Supplement 1). These results were observed for each cognitive test (except for the TMT-A among participants with mild HI).

**Figure.**
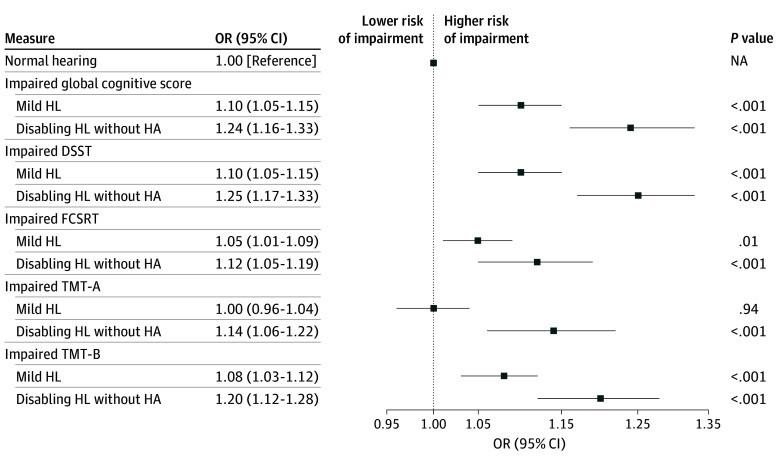
Association Between Hearing Status and the Individual Scores of Cognitive Impairment Analyses were performed among participants without hearing aids (HAs) (n = 60 404). Models were adjusted for sex, age, body mass index, lifetime noise exposure, social and personal deprivation, educational level, diabetes, prevalent cardiovascular disease, hypertension, depression, smoking status, and alcohol consumption. Impairment was defined by a score less than or equal to 25% of the total population’s score for global cognitive impairment and by a score less than or equal to 25% of the norms defined in the CONSTANCES cohort, adjusted for sex, age, and educational level for the Digit Symbol Substitution Test (DSST) and Free and Cued Selective Reminding Test (FCSRT), and greater than or equal to 75% for the Trail Making Test (TMT). HL indicates hearing loss; NA, not applicable; OR, odds ratio; TMT-A, Trail Making Test to evaluate shifting abilities; TMT-B, Trail Making Test to evaluate executive functions.

### Association Between HA Use and Cognition

This analysis was conducted among the 7680 participants who either were HA users or had disabling HL without HA use. As described in Table 1, other than deprivation and level of education, the baseline characteristics did not differ substantially between participants without HA use and those with HA use. The proportion of global cognitive impairment was 34% in participants with HA use and 37% of participants without HA use. In multivariable analysis, the OR for cognitive impairment among participants with HA use vs no HA use was 0.94 (95% CI, 0.83-1.07). Consistent results were found when considering propensity score analyses, as summarized in Table 3.

**Table 3.** Odds of Cognitive Impairment Associated With Hearing Aid Use^a^

Variable	OR (95% CI)
Multivariable analysis	0.94 (0.83-1.07)
Use of propensity score	
Adjusted on PS (continuous)	0.95 (0.85-1.07)
Matched on PS	0.99 (0.86-1.14)
IPTW	0.94 (0.85-1.04)

Abbreviations: IPTW, inverse probability of treatment weighting; OR, odds ratio; PS, propensity score.

^a^ Analyses were performed among 7680 individuals (hearing aid: 1668, disabling hearing loss, 6012). The multivariable analysis was adjusted for sex, age, personal deprivation, lifetime noise exposure, social deprivation, body mass index, educational level, diabetes, prevalent cardiovascular disease, hypertension, depression, smoking status, and alcohol consumption.

### Sensitivity Analyses

First, when considering a global cognitive score constructed from all 5 tests (including MMSE and VFT scores and the 3 remaining subtests of the FCSRT), the proportion of cognitive impairment increased with the level of HL (eTable 3 in Supplement 1), as confirmed by the multivariable analysis (eTable 4 in Supplement 1), aligning with the results of the main analysis. No trends were observed when considering the delayed and cued sections of the FCSRT because of a threshold effect of these scores. Second, the findings remained consistent when evaluating global cognition as a continuous score (eTable 5 in Supplement 1). Third, while observed in participants with and those without depression, the ORs between HL and global cognitive impairment were greater among the former (eTable 6 in Supplement 1). An association between HA use and global cognitive impairment was seen among participants with depression (OR, 0.62; 95% CI, 0.44-0.88) (eTable 6 in Supplement 1), but not among those without depression (*P* = .02 for interaction). After multiple imputations, the associations between HL and global cognitive impairment and the lack of association between HA use and global cognitive impairment remained (eTable 7 in Supplement 1). Third, the ORs remained globally unchanged after adjustment on hearing test condition (OR, 1.11; 95% CI, 1.06-1.16 for mild HL; OR, 1.25; 95% CI, 1.17-1.34 for disabling HL).

## Discussion

From this large population-based cohort study, HL was consistently associated with cognitive impairment as assessed by 5 cognitive tests among adults aged 45 to 69 years, in a graded manner. Hearing aid use was not associated with a significantly lower odds of cognitive impairment, except in participants with depression, compared with those with disabling HL without HA use.

In 2020, the *Lancet* Commission stated that HL constitutes the largest contributing factor to cognitive decline worldwide, accounting for 8% of dementia.^2^ This is supported by the results of 2 meta-analyses of observational studies showing a doubling of risk for either incident dementia or cognitive impairment among individuals with HL compared with those without.^48,49^ However, the first meta-analysis only included 3 prospective studies totaling less than 3000 participants (mean age, 67 years; range, 36-90 years), and the second considered only 5 cross-sectional studies totaling less than 7000 participants (mean age range, 71-85 years). Furthermore, in the second meta-analysis, audiometric assessment was highly heterogeneous between studies; ranging from evaluating only 2 frequencies to examining the entire spectrum of the frequencies or considering either the better or the worst ear. By evaluating the entire spectrum of frequencies in both ears, as well as several dimensions of cognition, the present study addresses many of these gaps. Some studies examined the association between objectively measured HL and dementia. For instance, an observational study including 2413 participants found a higher prevalence ratio of dementia in participants with moderate to severe HL (1.61; 95% CI, 1.09-2.38) compared with participants with normal hearing.^26^ Moreover, a recent Danish population-based study conducted among adults aged 50 years and older reported an association between objectively measured severe HL and incident dementia as assessed through national registries, with a hazard ratio (HR) of 1.20 (95% CI, 1.09-1.32).^50^ The present findings showing an OR of 1.24 of disabling HL for global cognitive impairment are consistent with the results of the Danish study on dementia.

Evidence on the possible benefit of HA use on cognition remains controversial. Two observational studies have addressed this question.^50,51^ An analysis using data from the UK Biobank initially reported a lower risk of dementia in participants with HAs compared with those without HAs, but the article was thereafter retracted due to a switch in group exposure.^51^ In the previously mentioned Danish study,^50^ the authors concluded positively on the benefit of HAs to prevent dementia. Compared with participants without HL, those with HL who were using HAs had a lower risk of dementia than those with HL but without HA use (HR, 1.06; 95% CI, 1.01-1.10 vs HR, 1.20; 95% CI, 1.13-1.27). However, to evaluate the potential benefit of HA use, analyses should be conducted among participants with HL, hence evaluating the effect of HA use in itself, which was not done in the abovementioned study^50^ as in others.^13^ The results of the present study comparing the use of HAs among participants with HL are not in favor of a clear benefit of HA on cognition. However, drawing conclusions on the benefit of HAs from meta-analyses remains difficult given the huge heterogeneity of the included studies. For instance, the latest meta-analysis on this topic found that HA use was associated with a lower hazard of cognitive decline.^24^ However, this result was obtained from a pooled analysis of 8 studies, among which 6 found no association between HA use and cognition, and the result was mainly observed in 1 study (weight, 70%) in which HL, HA, and Alzheimer disease data were obtained through administrative codes.^52^ Moreover, the 8 included studies were heterogeneous regarding the evaluated device (HAs or cochlear implants), the levels of HL, and the cognitive evaluations.

To our knowledge, only 1 randomized clinical trial has evaluated the benefit of HA use on cognition.^25^ In 977 US older adults (age, 70-84 years) with initially untreated HL and without substantial cognitive decline, HA use was unrelated to a significant neurocognitive change over 3 years.^25^ In a subgroup analysis (n = 238) of patients at higher risk of cognitive decline and dementia (ie, older patients, more CVD risk factors, lower baseline cognitive scores, and faster rates of 3-year cognitive decline than the rest of the cohort), HA use was associated with a 48% risk reduction of cognitive decline. Our analysis extends these study findings to a much younger population. Similarly to this randomized clinical trial, we observed that HA use was associated with less cognitive impairment in a subgroup of vulnerable individuals: those with depression.^46^ A synergistic beneficial association of HA use with social isolation caused by both depression and auditory deprivation might explain this finding.

The potential mechanisms underpinning the association between HL and cognitive impairment encompass social isolation stemming from both hearing and cognitive impairments,^5^ cognitive resource redistribution toward auditory perceptual processing,^53^ cognitive decline resulting from prolonged auditory input deprivation, and shared neurodegenerative processes in the aging brain associated with both cognitive deterioration and HL.^54,55,56,57,58,59^ Additionally, HL has been identified as a risk factor for temporal lobe volume loss and decline in the hippocampus and entorhinal cortex.^59,60^

Altogether, the present findings combined with prior evidence support that patients with HL are at higher risk of cognitive impairment. However, evidence is currently lacking to support screening for cognitive impairment in adults,^61^ and this is likely to also apply to the presently examined population: middle-aged adults diagnosed with HL. Furthermore, HA prescription in patients with disabling HL should be guided by their established benefits on quality of life and social isolation,^62,63^ but not to mitigate cognitive decline for which further research is needed.

### Strengths and Limitations

Strengths of the study include the size of the analytic sample, the use of objective measures of hearing, multiple cognitive tests reflecting several dimensions of cognition, and the consistency of the study findings on sensitivity analysis. The study has limitations. First, of 85 885 eligible participants, 23 813 were excluded due to missing data on audition, cognition, and covariates. However, consistent associations were found in sensitivity analyses including multiple imputations, making selection bias unlikely. Second, the cross-sectional design of the present study limits interpretation of the associations between HL, HA use, and cognitive impairment. Moreover, hearing and cognitive evaluations took place at the same time. Third, data such as the duration of HA use by participants, the mean number of hours per day with HA use, and whether HA use preceded the onset of cognitive impairment were unavailable. Fourth, despite accounting for several covariates, residual confounding cannot be excluded. Fifth, hearing level was not assessed in participants with HAs, and we cannot exclude that some participants may use unilateral HAs with a contralateral ear having normal hearing. Sixth, despite adjustment on several markers of socioeconomic status, we cannot rule out any bias related to the socioeconomic status in the acquisition and use of HAs. Seventh, the analysis was conducted in a mostly White population so that the results may not apply to other racial or ethnic groups.

## Conclusions

The findings of this cohort study suggest a robust association between HL and cognitive impairment. However, no association between HA use and cognitive impairment was noted.

## Notes

Supplement 1. **eTable 1**. Characteristics of Included and Excluded Participants **eTable 2**. Odds Ratios of global Cognitive Impairment Associated With Hearing Status: Results for the Covariates **eTable 3**. Participants’ Cognitive Performances on Supplementary Scores by Hearing Status. **eTable 4**. Association Between Cognitive Impairment as Assessed by MMSE, FCSRT and VFT, and Hearing Status **eTable 5**. Association Between Global Cognitive Score Considered as a Continuous Variable, Hearing Status and Hearing Aid Use **eTable 6.** Association Between Global Cognitive Impairment, Hearing Status, and Hearing Aid Use, Stratified by Depression Status **eTable 7.** Association Between Global Cognitive Impairment, Hearing Status, and Hearing Aid Use, After Multivariable Imputation by Chained Equations **eFigure 1**. Principal Component Analysis for the Global Cognitive Score **eFigure 2**. Study Flowchart **eFigure 3**. Proportion of Global Cognitive Impairment by Hearing Status and Age Category

Supplement 2.
Data Sharing Statement
