# Supplementary material for: Hearing Loss, Hearing Aids, and Cognition
Source: JAMA Netw Open. 2024 Oct 1;7(10):e2436723. doi: 10.1001/jamanetworkopen.2024.36723 (PMC11445684; doi:10.1001/jamanetworkopen.2024.36723)
Supplement: Supplement 1. — eTable 1. Characteristics of Included and Excluded Participants eTable 2. Odds Ratios of global Cognitive Impairment Associated With Hearing Status: Results for the Covariates eTable 3. Participants’ Cognitive Performances on Supplementary Scores by Hearing Status. eTable 4. Association Between Cognitive Impairment as Assessed by MMSE, FCSRT and VFT, and Hearing Status eTable 5. Association Between Global Cognitive Score Considered as a Continuous Variable, Hearing Status and Hearing Aid Use eTable 6. Association Between Global Cognitive Impairment, Hearing Status, and Hearing Aid Use, Stratified by Depression Status eTable 7. Association Between Global Cognitive Impairment, Hearing Status, and Hearing Aid Use, After Multivariable Imputation by Chained Equations eFigure 1. Principal Component Analysis for the Global Cognitive Score eFigure 2. Study Flowchart eFigure 3. Proportion of Global Cognitive Impairment by Hearing Status and Age Category [file jamanetwopen-e2436723-s001.pdf]

Supplementary Online Content

Grenier B, Berr C, Goldberg M, et al. Hearing loss, hearing aids, and cognition. *JAMA Netw Open*. 2024;7(10):e2436723. doi:10.1001/jamanetworkopen.2024.36723

eTable 1. Characteristics of Included and Excluded Participants

eTable 2. Odds Ratios of global Cognitive Impairment Associated With Hearing Status: Results for the Covariates

eTable 3. Participants' Cognitive Performances on Supplementary Scores by Hearing Status.

eTable 4. Association Between Cognitive Impairment as Assessed by MMSE, FCSRT and VFT, and Hearing Status

eTable 5. Association Between Global Cognitive Score Considered as a Continuous Variable, Hearing Status and Hearing Aid Use

eTable 6. Association Between Global Cognitive Impairment, Hearing Status, and Hearing Aid Use, Stratified by Depression Status

eTable 7. Association Between Global Cognitive Impairment, Hearing Status, and Hearing Aid Use, After Multivariable Imputation by Chained Equations

eFigure 1. Principal Component Analysis for the Global Cognitive Score

eFigure 2. Study Flowchart

eFigure 3. Proportion of Global Cognitive Impairment by Hearing Status and Age Category

This supplementary material has been provided by the authors to give readers additional information about their work.

eTable 1: Characteristics of included and excluded participants.

	Included<br>N= 62 072	Excluded<br>N= 23 813	*p-value*
**General characteristics**			
Male sex	29 832 (48)	10 719 (55)	< 0.001
Age, *years, mean ± SD*	57.4 ± 7	58.4 ± 7	< 0.001
Body mass index, *mean ± SD*	25.7 ± 4	26 ± 5	< 0.001
Noise exposure, *N (%)*	18 682 (30)	6 318 (31)	< 0.001
**Socio-professional**			
Social deprivation, *N (%)*	12 129 (20)	4 551 (19)	0.25
Personal deprivation, *N (%)*	8 652 (14)	5 891 (25)	< 0.001
Diploma, *N (%)*			< 0.001
Primary education	1 617 (3)	1 371 (6)	
Lower secondary education	4 795 (8)	2 325 (11)	
Upper secondary education	24 097 (39)	8 184 (38)	
Bachelor's degree	20 413 (33)	5 927 (28)	
Master's or doctorate degree	11 150 (18)	3 632 (17)	
**Comorbidities**			
Diabetes, *N (%)*	2 272 (4)	1 152 (5)	< 0.001
Prevalent CVD, *N (%)*	2 150 (3)	979 (4)	< 0.001
Hypertension, *N (%)*	11 183 (18)	5 331 (23)	< 0.001
Depression, *N (%)*	7 765 (13)	2 900 (17)	< 0.001
Smoking at baseline, *N (%)*			< 0.001
Never	27 688 (45)	9 202 (48)	
Past	26 384 (43)	2 779 (38)	
Current	8 000 (13)	7 384 (14)	
Alcohol consumption, *N (%)*			< 0.001
Never	2 447 (4)	2 137 (11)	
Low Risk	49 113 (79)	14 146 (72)	
Harmful	8 371 (13)	2 578 (13)	
Dependence	2 141 (3)	830 (4)	
**Audition**		*n = 19 216*	
PTA, *mean ± SD, dB HL*	22.1 ± 11	23.5 ± 12	< 0.001
All hearing loss, *N (%)*	31 448 (51)	10 117 (42)	< 0.001
Mild hearing loss, *N (%)*	23 768 (38)	7 776 (33)	< 0.001
Disabling hearing loss, *N (%)*	6 012 (10)	2 341 (6)	< 0.001
Hearing aid use, *N (%)*	1 722 (3)	613 (3)	0.64

Abbreviations: CVD: cardiovascular disease; dB HL: decibels hearing level; PTA: pure-tone average; SD: standard deviation.

© 2024 Grenier et al. *JAMA Network Open.*

eTable 2: Odds ratios of global cognitive impairment associated with hearing status: results for the covariates.

	**Model 1**<br>Odds Ratio [95% CI]	**Model 2**<br>Odds Ratio [95% CI]
**Hearing Status**		
Normal hearing	1 [*reference*]	1 [*reference*]
Mild hearing loss	1.13 [1.07 - 1.18]	1.10 [1.05 - 1.15]
Disabling hearing loss	1.32 [1.23 - 1.41]	1.24 [1.16 - 1.33]
**General characteristics**		
Sexe		
Male	1 [*reference*]	1 [*reference*]
Female	0.49 [0.47 - 0.51]	0.48 [0.46 - 0.51]
Age*, per year*	1.10 [1.09 - 1.10]	1.10 [1.10 - 1.11]
Diploma		
Primary education	1 [*reference*]	1 [*reference*]
Lower secondary education	0.34 [0.30 - 0.39]	0.38 [0.34 - 0.43]
Upper secondary	0.26 [0.23 - 0.29]	0.31 [0.27 - 0.34]
Bachelor's degree	0.11 [0.10 - 0.12]	0.15 [0.13 - 0.16]
Master's or doctorate degree	0.07 [0.06 - 0.08]	0.10 [0.09 - 0.11]
Body mass index, *per point*	- -	1.00 [1.00 - 1.01]
Noise exposure lifetime	- -	1.35 [1.29 - 1.42]
Social deprivation	- -	1.03 [0.98 - 1.09]
Personal deprivation	- -	1.68 [1.59 - 1.78]
**Comorbidities**		
Diabetes	- -	1.12 [1.01 - 1.24]
Prevalent CVD	- -	1.12 [1.01 - 1.23]
Hypertension	- -	1.11 [1.05 - 1.17]
Depression	- -	1.38 [1.30 - 1.47]
Smoking at baseline		
Never	- -	1 [*reference*]
Past	- -	0.78 [0.74 - 0.81]
Current	- -	0.98 [0.91 - 1.05]
Alcohol consumption		
Never	- -	1 [*reference*]
Low Risk	- -	0.56 [0.41 - 0.62]
Harmful	- -	0.51 [0.45 - 0.57]
Dependence	- -	0.57 [0.49 - 0.66]

Abbreviations: CI: confidence interval; CVD: cardiovascular disease.
Note: Analyses are performed among non-hearing aid users only *(n = 60 404).* Model 1 is a logistic regression adjusted for sex, age and education level. Model 2 is additionally adjusted for lifetime noise exposure, social and personal deprivation, BMI, diabetes, prevalent cardiovascular disease, hypertension, depression, smoking status and alcohol consumption.
Global cognitive impairment is defined by a score ≤ 25% of the total population's score.

© 2024 Grenier et al. *JAMA Network Open.*

eTable 3: Participants' cognitive performances on supplementary scores by hearing status.

	Total population	Normal hearing	Hearing loss			
			All HL	Mild HL	Disabling HL without HA	Hearing aid user
**Audition**						
PTA, *mean* ±*SD, dB HL*	22.1 ± 11	14.2 ± 4	30.3 ± 9	26.6 ± 4	45.0 ±10	- -
**Global cognition (5 tests)**						
Global cognitive score, *mean* ± *SD*	- 0.13 ± 2.2	0.44 ± 1.6	0.28 ± 1.7	- 0.11 ± 1.7	- 0.62 ± 1.8	-0.38 ± 1.7
Global cognitive impairment	14 242 (23)	5 050 (16)	9 160 (29)	6 337 (27)	2 272 (38)	551 (33)
**Mini-Mental State Examination**						
MMSE score, *mean* ± *SD*	28.2 ± 2	28.4 ± 1.6	28.1 ± 2	28.1 ± 2	27.9 ± 2	28 ± 2
MMSE impairment	21 294 (34)	9 761 (32)	11 533 (37)	8 468 (36)	2 409 (40)	656 (39)
**Free and Cued Selective Reminding Test**						
FCSRT2 [a] score, *mean* ± *SD*	46.4 ± 3	46.4 ± 3	46.1 ± 3	46.2 ± 3	45.9 ± 3	46 ± 3
FCSRT2 impairment	20 956 (34)	10 476 (34)	10 480 (33)	7 994 (34)	1 942 (32)	544 (33)
FCSRT3 [b] score, *mean* ± *SD*	12.7 ± 2	13.0 ± 2	12.4 ± 2	12.5 ± 2	12.1 ± 3	12.2 ± 2
FCSRT3 impairment	20 861 (34)	10 091 (33)	10 770 (34)	8 054 (34)	2 116 (35)	600 (36)
FCSRT4 [c] score, *mean* ± *SD*	15.6 ± 1	15.7 ± 1	15.6 ± 1	15.6 ± 1	15.5 ± 1	15.6 ± 2
FCSRT4 impairment	49 508 (80)	25 941 (85)	23 567 (75)	18 240 (77)	4 115 (68)	1 212 (73)
**Verbal Fluency Test**						
VFT-A score, *mean* ± *SD*	23.8 ± 6	24.2 ± 6	23.4 ± 6	23.5 ± 6	22.8 ± 6	23.5 ± 6
VFT-A impairment	16 222 (26)	7 854 (26)	8 368 (27)	6 228 (26)	1 718 (29)	422 (25)
VFT-B score, *mean* ± *SD*	15.2 ± 5	15.7 ± 5	14.8 ± 5	15 ± 5	14.1 ± 5	14.6 ± 5
VFT-B impairment	18 237 (26)	8 677 (28)	9 560 (30)	7 039 (30)	1 998 (33)	523 (31)

Abbreviation: dB HL, decibels hearing level; HL, hearing loss; PTA, pure Tone Average; SD, standard deviation; HA: hearing aids.
Note: impairment is defined by a score ≤ 25% of the total population's score for global cognitive impairment and by a score ≤ 25% of the norms defined in the Constances cohort, adjusted for sex, age and education for DSST and FCSRT, and ≥ 75% for TMT.
[a] FCSRT2 represent the cued immediate recall. [b] FCSRT3 represent the free delayed recall. [c] FCSRT4 represent the cued delayed recall.

© 2024 Grenier et al. *JAMA Network Open.*

eTable 4: Association between cognitive impairment as assessed by MMSE, FCSRT and VFT, and hearing status.

	**Normal Hearing**	**Mild HL**<br>Odds Ratio [CI 95%]	**Disabling HL without HA**<br>Odds Ratio [CI 95%]
**Global impairment** (5 tests)	1 [*Ref.*]	1.13 [1.07 - 1.18]	1.34 [1.25 - 1.43]
**Mini-Mental State Examination**			
MMSE impairment	1 [*Ref.*]	1.13 [1.09 - 1.17]	1.32 [1.24 - 1.40]
**Free and Cued Selective Reminding Test**			
FCSRT2 [a] impairment	1 [*Ref.*]	1.04 [1.00 - 1.08]	1.04 [0.97 - 1.10]
FCSRT3 [b] impairment	1 [*Ref.*]	1.02 [0.98 - 1.06]	1.06 [1.00 - 1.13]
FCSRT4 [c] impairment	1 [*Ref.*]	1.03 [0.96 - 1.10]	1.13 [1.03 - 1.25]
**Verbal Fluency Test**			
VFT-A impairment	1 [*Ref.*]	1.03 [0.98 - 1.07]	1.14 [1.06 - 1.21]
VFT-B impairment	1 [*Ref.*]	1.04 [1.00 - 1.08]	1.20 [1.12 - 1.27]

Abbreviation: CI: confidence interval; HL: hearing loss; *Ref*: reference; HA: hearing aids.
Note: Analyses are performed among non-hearing aid users only *(n = 60 404).* Models are logistic regressions adjusted for sex, age, BMI, lifetime noise exposure, social and personal deprivation, education level, diabetes, prevalent cardiovascular disease, hypertension, depression, smoking status and alcohol consumption. Impairment is defined by a score ≤ 25% of the total population's score for global cognitive impairment and by a score ≤ 25% of the norms defined in the Constances cohort, adjusted for sex, age and education for DSST and FCSRT, and ≥ 75% for TMT.
[a] FCSRT2 represent the cued immediate recall. [b] FCSRT3 represent the free delayed recall. [c] FCSRT4 represent the cued delayed recall.

© 2024 Grenier et al. *JAMA Network Open.*

eTable 5: Association between global cognitive score considered as a continuous variable, hearing status and hearing aid use.

	Model 1		Model 2	
	Coefficient	*p-value*	Coefficient	*p-value*
**Hearing Status (n=60 404)**				
Normal hearing	*reference*	-	*reference*	-
Mild hearing loss	- 0.08	*<0.001*	- 0.06	*<0.001*
Disabling hearing loss without hearing aids	- 0.22	*<0.001*	- 0.17	*<0.001*
**Hearing Aid Status (n=7 680)**				
Non-user	*reference*	-	*reference*	-
User	+ 0.14	< *0.001*	+ 0.07	*0.07*

Note: model 1 is a linear regression adjusted for sex, age and education level. Model 2 is additionally adjusted for lifetime noise exposure, social and personal deprivation, BMI, diabetes, prevalent cardiovascular disease, hypertension, depression, smoking status and alcohol consumption. The analysis on hearing status is conducted among those without hearing aids. The analysis on hearing aids status is conducted among those with disabling hearing loss without HA and those with HA.

© 2024 Grenier et al. *JAMA Network Open.*

eTable 6: Association between global cognitive impairment, hearing status, and hearing aid use, stratified by depression status.

	Non-depressed participants<br><br>Odds Ratio [CI 95%]	Depressed participants<br><br>Odds Ratio [CI 95%]
**Hearing Status**	***N=52 864***	***N=7 540***
Normal hearing	1 [*reference*]	1 [*reference*]
Mild hearing loss	1.08 [1.03 - 1.14]	1.19 [1.06 - 1.35]
Disabling hearing loss without hearing aids	1.22 [1.13 - 1.32]	1.37 [1.14 - 1.64]
**Hearing Aid Status**	***N=6 650***	***N=1 030***
Non-user	1 [*reference*]	1 [*reference*]
User	1.0 [0.88 - 1.15]	0.62 [0.44 - 0.88]

Abbreviations: CI: confidence interval.
Note: regression models are performed among participants withour hearing aids and are adjusted for sex, age, social and personal deprivation, lifetime noise exposure, BMI, education level, diabetes, prevalent cardiovascular disease, hypertension, smoking status and alcohol consumption. The analysis on hearing status is conducted among those without hearing aids. The analysis on hearing aids status is conducted among those with disabling hearing loss without HA and those with HA.

© 2024 Grenier et al. *JAMA Network Open.*

eTable 7: Association between global cognitive impairment, hearing status, and hearing aid use, after multivariable imputation by chained equations.

	Model 1<br>Odds Ratio [CI 95%]	Model 2<br>Odds Ratio [CI 95%]
**Hearing Status (N=75 058)**		
Normal hearing	1 [*reference*]	1 [*reference*]
Mild hearing loss	1.24 [1.19 - 1.29]	1.08 [1.04 - 1.13]
Disabling hearing loss without hearing aids	1.59 [1.50 - 1.68]	1.21 [1.14 - 1.28]
**Hearing Aid Status (N=9 906)**		
Non-user	1 [*reference*]	1 [*reference*]
User	0.85 [0.77 - 0.93]	0.93 [0.83 - 1.03]

Abbreviations: CI: confidence interval.
Note: due to the imputation process, the sample size of the hearing status stratum (n=75 058) and of the hearing aids status stratum (n=9 906) is greater than in the main analysis. Regression models are performed among participants withour hearing aids. Model 1 is adjusted for sex, age and education level for Hearing Status. Model 2 is additionally adjusted for lifetime noise exposure, social deprivation, education level or personnal deprivation, BMI, diabetes, prevalent cardiovascular disease, hypertension, depression, smoking status, and alcohol consumption. The analysis on hearing status is conducted among those without hearing aids. The analysis on hearing aids status is conducted among those with disabling hearing loss without HA and those with HA.

© 2024 Grenier et al. *JAMA Network Open.*

eFigure 1: Principal component analysis for the global cognitive score.

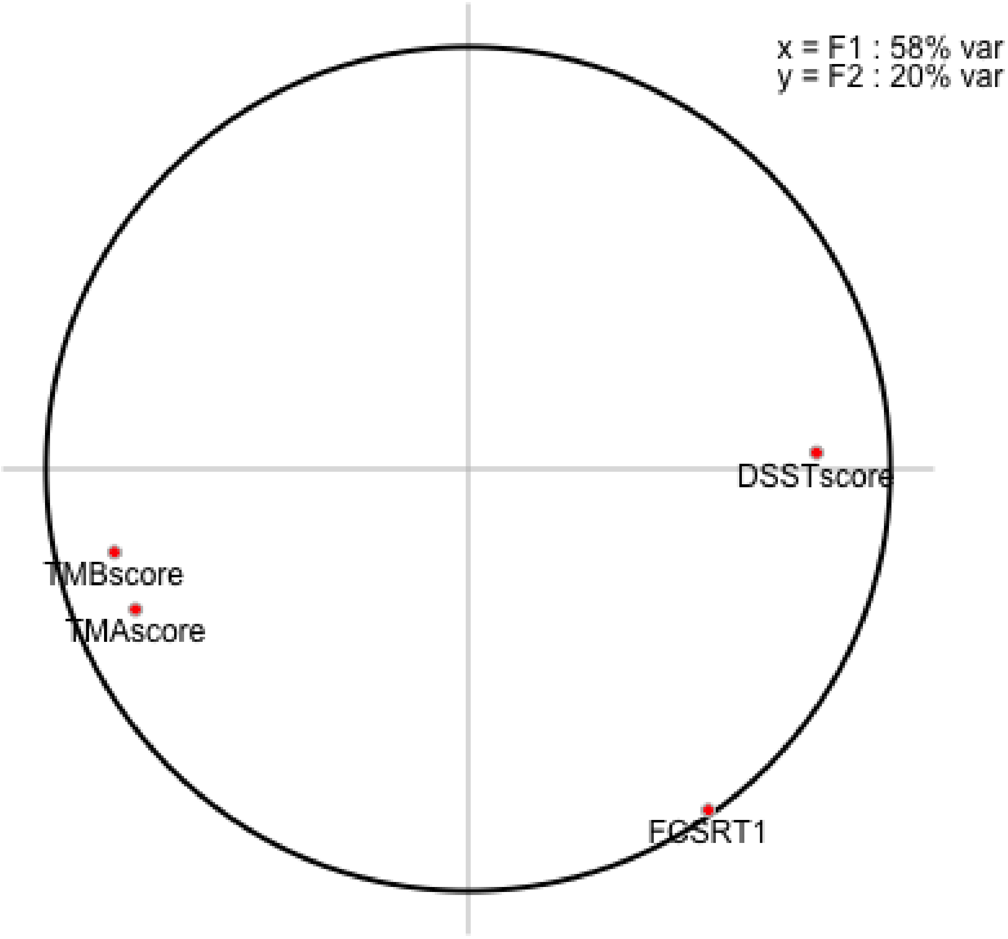

Abbreviations: DDST: digit symbol substition test; FCSRT: free and cued selective reminding test; TMA/TMB: trail making test.
Note: FCSRT represent the free immediate recall.

© 2024 Grenier et al. *JAMA Network Open.*

eFigure 2: Study flowchart.

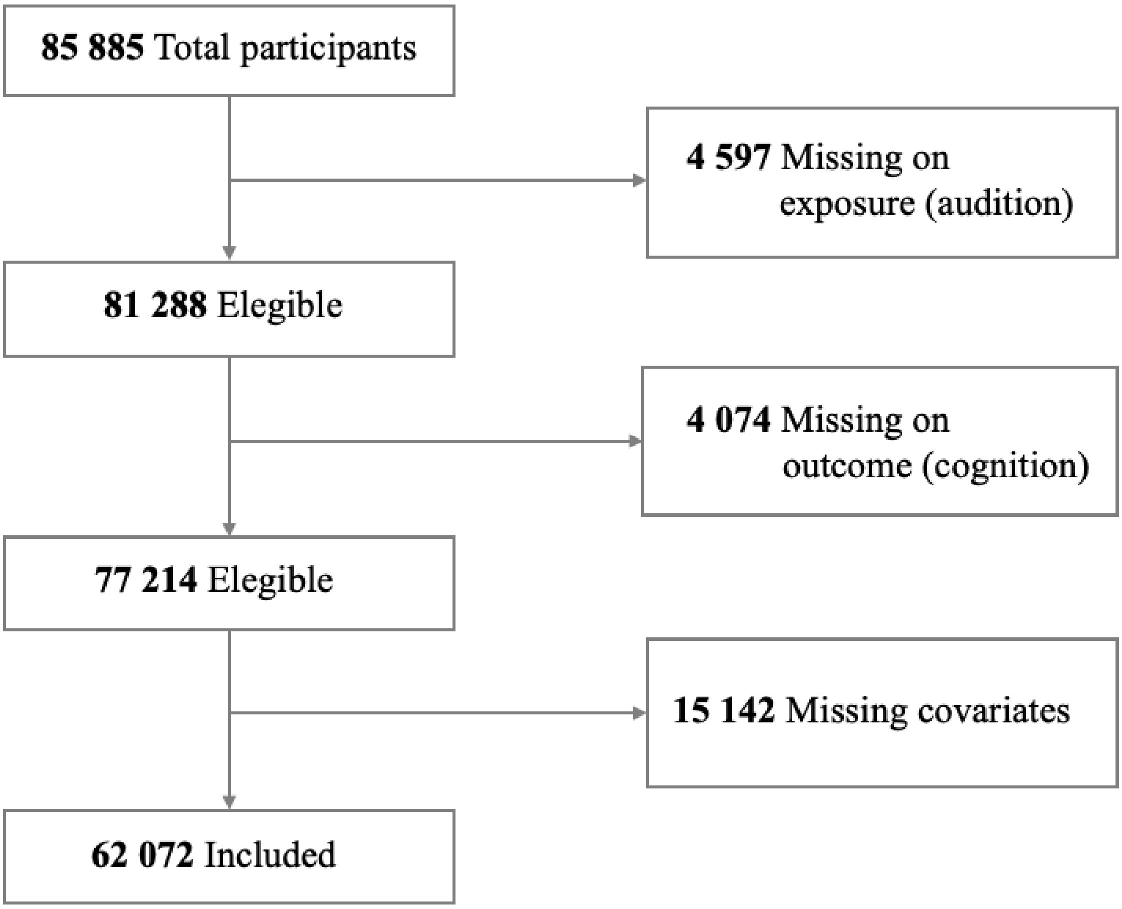

© 2024 Grenier et al. *JAMA Network Open.*

eFigure 3: Proportion of global cognitive impairment by hearing status and age category.

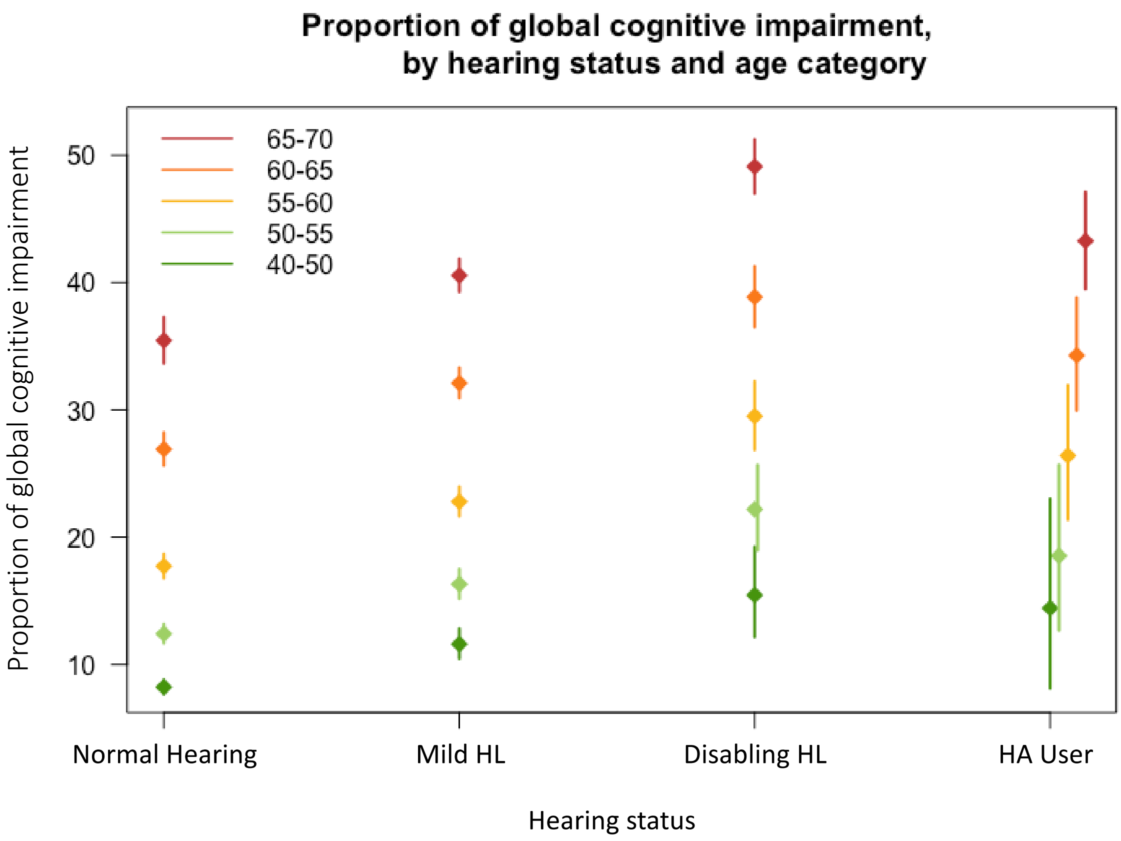

Abbreviation: HA: hearing aid; HL: hearing loss.
Note: Vertical lines represent 95% confidences intervals.

© 2024 Grenier et al. *JAMA Network Open.*
