# Supplementary material for: Hearing Loss, Hearing Aids, and Cognition
Source: JAMA Netw Open. 2024 Oct 1;7(10):e2436723. doi: 10.1001/jamanetworkopen.2024.36723 (PMC11445684; doi:10.1001/jamanetworkopen.2024.36723)
Supplement: Supplement 2. — Data Sharing Statement [file jamanetwopen-e2436723-s002.pdf]

Data Sharing Statement

Grenier. Hearing Loss, Hearing Aids, and Cognition. *JAMA Netw Open*. Published October 01, 2024. doi:10.1001/jamanetworkopen.2024.36723

Data
Data available: No
